# Translational Pharmacokinetic–Pharmacodynamic Modeling of NaV1.7 Inhibitor MK-2075 to Inform Human Efficacious Dose

**DOI:** 10.3389/fphar.2021.786078

**Published:** 2021-12-24

**Authors:** Jeanine E. Ballard, Parul S. Pall, Joshua Vardigan, Fuqiang Zhao, Marie A. Holahan, Xiaoping Zhou, Nina Jochnowitz, Richard L. Kraus, Rebecca M. Klein, Darrell A. Henze, Andrea K. Houghton, Christopher S. Burgey, Christopher Gibson, Arie Struyk

**Affiliations:** ^1^ Pharmacokinetics Pharmacodynamics and Drug Metabolism, Merck & Co. Inc., Kenilworth, NJ, United States; ^2^ Neuroscience Pharmacology, Merck & Co. Inc., Kenilworth, NJ, United States; ^3^ Translational Imaging Biomarkers, Merck & Co. Inc., Kenilworth, NJ, United States; ^4^ Discovery Chemistry, Merck & Co. Inc., Kenilworth, NJ, United States; ^5^ Translational Medicine, Merck & Co. Inc., Kenilworth, NJ, United States

**Keywords:** NaV1.7, nociception, pain, modeling, PKPD, MK-2075

## Abstract

MK-2075 is a small-molecule selective inhibitor of the NaV1.7 channel investigated for the treatment of postoperative pain. A translational strategy was developed for MK-2075 to quantitatively interrelate drug exposure, target modulation, and the desired pharmacological response in preclinical animal models for the purpose of human translation. Analgesics used as a standard of care in postoperative pain were evaluated in preclinical animal models of nociceptive behavior (mouse tail flick latency and rhesus thermode heat withdrawal) to determine the magnitude of pharmacodynamic (PD) response at plasma concentrations associated with efficacy in the clinic. MK-2075 was evaluated in those same animal models to determine the concentration of MK-2075 required to achieve the desired level of response. Translation of MK-2075 efficacious concentrations in preclinical animal models to a clinical PKPD target in humans was achieved by accounting for species differences in plasma protein binding and *in vitro* potency against the NaV1.7 channel. Estimates of human pharmacokinetic (PK) parameters were obtained from allometric scaling of a PK model from preclinical species and used to predict the dose required to achieve the clinical exposure. MK-2075 exposure–response in a preclinical target modulation assay (rhesus olfaction) was characterized using a computational PKPD model which included a biophase compartment to account for the observed hysteresis. Translation of this model to humans was accomplished by correcting for species differences in PK NaV1.7 potency, and plasma protein binding while assuming that the kinetics of distribution to the target site is the same between humans and rhesus monkeys. This enabled prediction of the level of target modulation anticipated to be achieved over the dosing interval at the projected clinical efficacious human dose. Integration of these efforts into the early development plan informed clinical study design and decision criteria.

## Introduction

A critical component of drug discovery and development is gaining an understanding of the relationship between drug exposure, target engagement or modulation at the site of action, and the desired pharmacological response (Bueters et al., 2015; Wong et al., 2017). The ability to interrelate these three components using a combination of clinical and preclinical information as early as possible in a drug discovery setting can increase the probability of achieving clinical success at a reasonable dose (Morgan et al., 2012). A recent analysis of the general correlation between *in vitro* potency and clinically efficacious unbound *in vivo* exposure has highlighted a variable relationship across therapeutic areas, target types, and mechanisms of action, and this emphasizes the importance of accounting for contributing factors such as target-specific pharmacology and turnover kinetics, drug distribution to the site of action, and *in vitro* assay conditions (Jansson-Löfmark et al., 2020). Therefore, it is important to develop a strategy to quantitatively integrate *in vitro* potency, exposure at the site of action, time course of biomarker response, and efficacy from preclinical models to predict clinical activity.

The objective of the work described herein was to develop a translational strategy for NaV1.7 inhibitor MK-2075 which would improve the likelihood of achieving therapeutically relevant target modulation in the clinic within the anticipated safety margins and to inform on the study design for pharmacodynamic (PD) endpoints. NaV1.7 has human genetic and preclinical validation for involvement in acute and chronic pain. Humans with genetic mutations leading to a loss of function (LOF) of the NaV1.7 channel have a congenital indifference to pain and anosmia while a gain of function mutations can result in pain syndromes such as primary erythromelalgia (Dib-Hajj et al., 2007; Weiss et al., 2011). Pharmacological inhibition of NaV1.7 channels in nonhuman primates has demonstrated a similar phenotype to humans with a genetic loss of NaV1.7 function, including hyposmia and analgesia (Kraus et al., 2021). While drugs with sodium channel–blocking activity such as carbamazepine have demonstrated some utility in pain treatment, their nonselective nature contributes to dose-limiting adverse effects (Tanelian and Victory, 1995; Dick et al., 2007; Moulin et al., 2014). MK-2075 is a small-molecule selective inhibitor of the NaV1.7 channel investigated for the potential treatment of postoperative pain.

**FIGURE 1:**
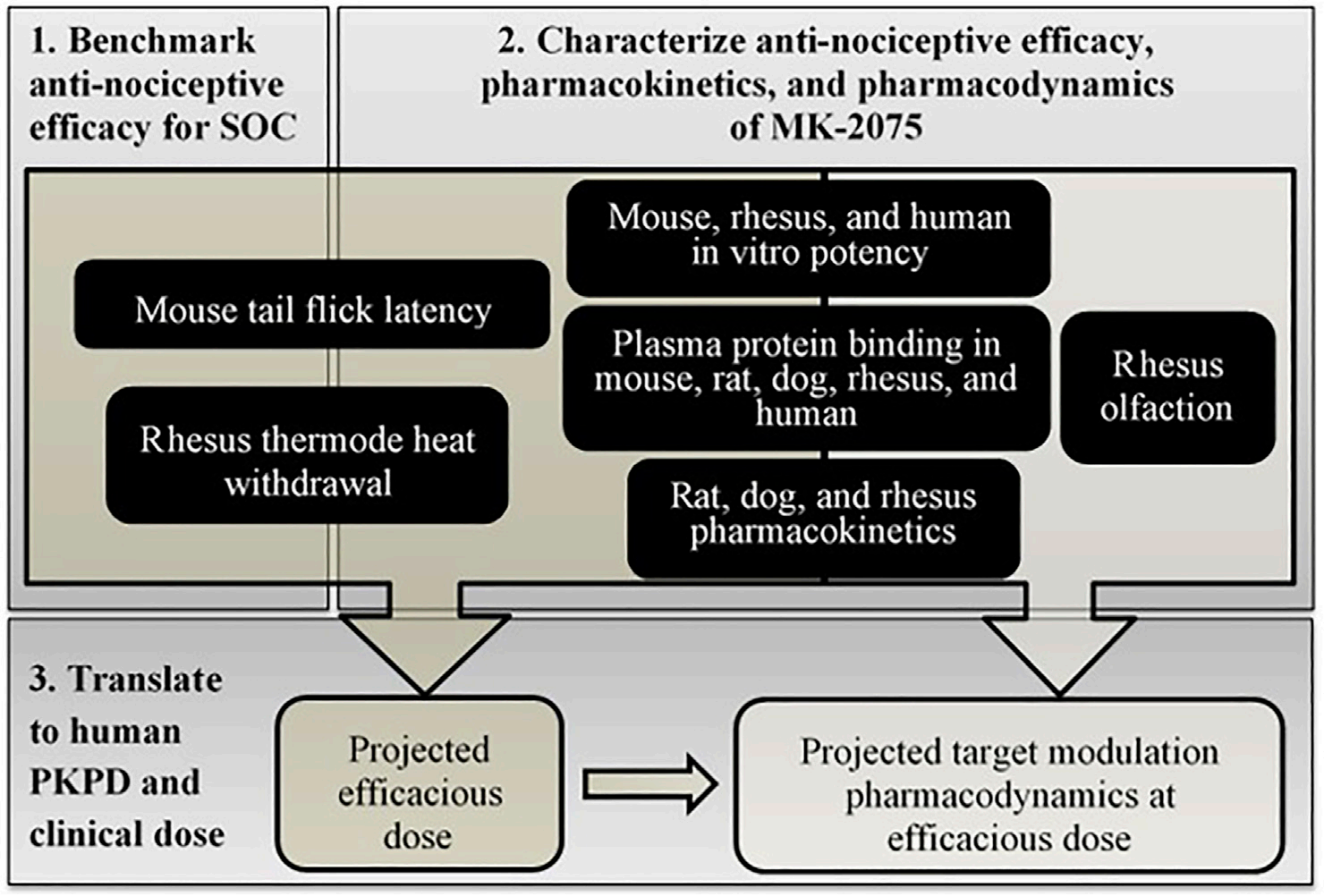
Schematic of step-wise workflow for translational PKPD of MK-2075 for prediction of dose and target modulation in humans.

The translational workflow for MK-2075 included benchmarking preclinical nociception assays with the clinical standard of care (SOC), characterization of MK-2075 preclinical PKPD and *in vitro* potency, scaling of preclinical pharmacokinetic (PK) and PD parameters to predict human clinical dose, and prediction of target modulation biomarker PD in humans at the anticipated efficacious dose (Figure 1). These efforts were integrated into a translational strategy enabling definition of the PKPD targets for MK-2075 in acute postoperative pain and prediction of efficacious human dose to achieve the PKPD target. The results of this work were used to identify a compound with reasonable probability of success to test inhibition of NaV1.7 as a mechanism for pain mitigation in the clinic. In addition, clinical assessment of olfaction was proposed to evaluate NaV1.7 target modulation in healthy volunteers, so translation of target modulation PD from rhesus olfaction measured by functional magnetic resonance imaging (fMRI) was used to inform early clinical study design and clinical decision criteria.

## Materials and Methods

### *In Vitro* NaV1.7 Potency

The intrinsic potency for inhibition of NaV1.7 from humans, rhesus monkeys, and mice was determined *in vitro*. Whole-cell sodium currents were recorded from a recombinant human embryonic kidney 293 cell line stably overexpressing either the human, mouse, or rhesus NaV1.7 channels using a manual patch clamp. Recording solutions comprised of the following (in mM): internal solution: 30 CsCl, 5 HEPES, 10 EGTA, 120 CsF, 5 NaF, 2 MgCl_2_, pH = 7.3 with CsOH; and external solution: 150 NaCl, 5 KCl, 2 CaCl_2_, 1 MgCl_2_, 10 HEPES, 12 dextrose, pH = 7.3 with NaOH. Pipettes were fabricated from borosilicate glass using a Sutter P-97 micropipette puller to an open tip resistance of 1–2 MΩ. Cells were voltage clamped at −60 mV for cell detection and sealing. At the start of the procedure, a voltage curve and an inactivation curve were run for each cell to determine the voltage at which 50% of the channels reside in the inactivated state (V_0.5inact_). The voltage curve was used to determine if the cell was adequately clamped; cells with currents larger than 10 nA or with space clamp issues were not used further. For the hyperpolarized state protocol, the holding potential was set to 20 mV negative of V_0.5inact_. A pulse train consisting of consecutive double pulses, an 8-s hyperpolarizing pre-pulse to −120 mV followed by a test pulse to −10 mV was applied at a frequency of 0.1 Hz. First, vehicle (0.3% DMSO) was added to establish a baseline measurement. MK-2075 was added after the baseline was established. Cells were exposed to MK-2075 for 5 min at a holding potential of 20 mV negative to V_0.5inact_ during which time no pulsing occurred. The cells were then exposed to the same hyperpolarized voltage protocol described above. A washout was performed to measure recovery of the sodium currents from inhibition. At least 3 concentrations of MK-2075 were tested with 3–5 replicates per concentration.

### *In Vitro* Plasma Protein Binding

Plasma protein binding was determined across species at a MK-2075 concentration of 1 or 2.5 µM using equilibrium dialysis against 100 mM PBS buffer with a 12–14 kDa threshold semipermeable membrane. After 4–6 h of incubation on a single-plate rotator at 37°C inside a CO_2_ chamber maintained at 5% CO_2_, aliquots of plasma and buffer were analyzed using LC-MS/MS. The fraction unbound in plasma (*f*_*u,p*_) was calculated as the ratio of MK-2075 concentration in buffer to that in plasma. Recovery of greater than 80% total MK-2075 from the assay was determined to confirm stability of the compound over the duration of the incubation.

### *In Vivo* Pharmacokinetics and Bioanalytical Methods

All animal studies were conducted according to the NIH Guide for the Care and Use of Laboratory Animals, and all protocols were reviewed and approved by the Institutional Animal Care and Use Committee at Merck & Co., Inc, Kenilworth, NJ, USA. The PK data for MK-2075 in the Wistar Han rat, beagle dog, and rhesus monkey were obtained following 2-h intravenous (IV) infusions. Doses administered were 1 mg/kg in 5% Captisol in rats, 0.4 mg/kg in 30% Captisol in dogs, and 2 mg/kg in 5% Captisol in monkeys. The dose levels in rats and monkeys were selected to achieve concentrations similar to the anticipated target clinical exposure based on the data from the preliminary low dose (0.05 mg/kg) IV bolus studies. Lower IV infusion doses were selected for dogs due to historical evidence of emesis and trembling with structural analogs of MK-2075. The blood samples were collected into EDTA tubes and centrifuged to obtain plasma prior to storage at −20°C until bioanalysis. The plasma samples were prepared for analysis using a protein precipitation technique, and the supernatant was analyzed by LC-MS/MS.

A two-compartment PK model with first-order elimination from the central compartment (differential equations (1) and (2)) was fit to preclinical plasma concentration–time data from rats, dogs, and monkeys separately in Phoenix 64 (Build 8.1.0.3530) to obtain the mean and standard error (SE) of the PK parameters *CL*, *V1*, *V2*, and *CLD*, as well as the correlation matrix for these parameters.

$$V1*\frac{dC}{dt}=\;-CL*C-CLD*C+CLD*C2$$ (1)

$$V2*\frac{dC2}{dt}=\;CLD*C-CLD*C2$$ (2)

where *CL* is the clearance from the central compartment, and *CLD* is the distributional clearance between the central and peripheral compartments. *V1* is the apparent volume of the central compartment, and *V2* is the apparent volume of the peripheral compartment. *C* is the concentration in the central compartment, and *C2* is the concentration in the peripheral compartment.

### Mouse Tail Flick Latency Methods and Modeling Strategy

*In vivo* antinociceptive activity was determined preclinically in a mouse tail flick latency study for two SOC agents for postoperative pain, morphine and tramadol, and the NaV1.7 inhibitor MK-2075 in a blinded and randomized design. Evaluation of antinociceptive activity was based on treatment-mediated increase in time to withdraw from a thermal stimulus. Briefly, BALB/c male mice were gently restrained and a focused infrared beam of radiant heat (Ugo Basilo, Italy) applied to a point approximately 2.5 cm from the tip of the tail. The intensity of the thermal stimulus was adjusted to provide an average baseline response of 3–5 s. The time taken for the mice to withdraw their tail from the heat stimulus was determined as the tail flick latency. To prevent tissue injury for the animals a cutoff time was set at 15 s. Mice not responding within the cutoff time were removed from the apparatus and assigned a latency of 15 s. After a baseline response was obtained, the mice were dosed (SC, in a volume of 10 ml/kg) with either vehicle or with the test compound. The tail flick responses were then measured at 40, 60, and 80 min after vehicle or compound administration. The drug concentration in the plasma was determined from the blood samples collected from the test animals at the end of the study (1–1.5 h postinjection).

The graphical analysis of the exposure–response relationship over time for a structural analog of MK-2075 showed no apparent hysteresis (data not shown). Therefore, a sigmoidal *E*_max_ model assuming a direct effect (Eq. 3) was fit to the tail flick latency measured at the last time point, either 60 or 80 min, as a function of the terminal total plasma concentration in Phoenix 64 (Build 8.1.0.3530) to estimate an *in vivo* potency (*EC*_50_) for MK-2075. The value of *E*_max_ was set equivalent to the difference between the latency threshold of 15 s (i.e., the maximum time for the application of the stimulus) and the model estimated baseline (*E*_0_).

$$E=E_{0}+\frac{{\text{E}}_{max}{\text{*C}}^{h}}{EC_{50^{h}}\;+\;C^{h}}$$ (3)

where, *E* is the total time to tail flick following thermal stimulation, *E*_0_ is the baseline tail flick latency in the absence of drug, and *E*_max_ is the maximum achievable effect level. *EC*_50_ is defined as the concentration required to increase the time to tail flick by 50% of the maximum achievable latency, *C* is the total plasma concentration measured at termination of the study, and *h* is the hill coefficient describing the steepness of the exposure–response relationship.

### Rhesus Thermode Heat Withdrawal Methods and Modeling Strategy

*In vivo* antinociceptive potency of MK-2075 was determined preclinically in the rhesus thermode heat withdrawal study based on treatment-mediated decrease in magnitude of behavioral response to a thermal stimulus on the forearm. Rhesus thermode heat withdrawal was measured by brief application of heat delivered to the forearm of rhesus macaque as described by Vardigan et al. (2018) and Kraus et al. (2021). Briefly, four test temperatures: 44°C, 46°C, 48°C, and 50°C were delivered randomly and repeated six times per session. The investigator rated the intensity of arm withdrawal evoked by heat stimuli on a scale from 0 to 2 (0: no movement, 1: a single movement, and 2: multiple movements of the test arm). The average response from the six replicate stimuli per temperature was reported. Experiments were performed 30 min following subcutaneous injection of vehicle or drug at two to four dose levels. Blood samples were collected 1 h following dosing for the measurement of drug concentration in the plasma.

An inhibitory *E*_max_ model assuming direct effect was fit to the heat response score data at a temperature of 46°C as a function of terminal total plasma concentration in Phoenix 64 (Build 8.1.0.3530):

$$E=E_{0}\text{* }1-\;\frac{C}{IC_{50}+C}$$ (4)

where *E* is the measured heat response score following thermal stimulation at 46°C, and *E*_0_ is the baseline response to thermal stimulus in the absence of drug. *C* is the total plasma concentration measured at the end of the study, and *IC*_50_ is defined as the concentration required to decrease the response score by 50%.

### Rhesus fMRI Olfaction Methods and Modeling Strategy

The detailed methods of fMRI of olfaction including animal preparation, experiment setup, anesthesia protocol, odor stimulation, MRI measurement, and data analysis have been described previously (Zhao et al., 2015). Briefly, odorant-induced olfaction in the olfactory bulb (OB) of anesthetized rhesus monkeys was monitored by multiple fMRI measurements made during a 4-h experiment session. Following 1 h of baseline measurement, MK-2075 was administered by IV route as a loading dose followed by 1 h of infusion at four dose levels. Multiple fMRI measurements of odorant-induced olfaction were made over the 1-h infusion period and 2-h washout period following termination of the infusion. Blood samples were collected at the end of the infusion for measurement of drug concentrations in the plasma by LC-MS/MS following protein precipitation.

Rhesus fMRI data was processed to determine percentage inhibition of olfaction over time (Ballard et al., 2020). Briefly, the time course of the fMRI signals was first obtained by averaging the time courses of all the activated pixels within the OB for each fMRI measurement, then the time courses from three consecutive fMRI measurements were averaged to yield one fMRI response for olfaction quantification. A total of 15 fMRI measurements performed during each 1-h block of the 4-h experiment session yielded 5 fMRI responses. A change in olfaction was expressed as percentage inhibition of the averaged fMRI responses following MK-2075 administration relative to the averaged fMRI responses during the 1-h baseline period before compound delivery.

Percentage inhibition of olfaction fMRI by NaV1.7 inhibitors as a function of exposure has been evaluated for time dependence in a previous publication (Ballard et al., 2020). The observed hysteresis was assumed to be a result of a distributional delay to the effect site, and a PKPD model with a biophase compartment (*Ce*) was utilized to account for the time delay between exposure and response.

While multiple measurements of olfaction inhibition by fMRI were obtained throughout the 4-h study period (∼12-min intervals), sampling for PK during the study was not feasible since the animal was isolated inside the fMRI machine. However, a single blood sample was collected from each animal at the end of the infusion to obtain a measure of the plasma concentration achieved in individual animals. In order to estimate the concentration–time profile over the entire study period in each individual animal, population PK parameters from a two-compartment model fit (Eqs. 1, 2) of MK-2075 concentration–time profile in the rhesus monkeys were determined from satellite IV infusion studies at 2 and 8 mg/kg (n = 3/dose). Post-hoc estimates of *CL*, *V1*, *V2*, and *CLD* were then obtained for individual subjects using population parameter estimates with interindividual variability from the population PK model anchored by the measured plasma concentration at the end of infusion for animals in the study.

The predicted exposure and observed response profile over time was used to fit a PD model with an effect-compartment in Phoenix 64 (Build 8.1.0.3530). The model structure contains a hypothetical compartment for concentrations at the site of effect with a first-order distribution rate constant *ke0* (Eq. 5). The PD model structure is a modification of the *E*_max_ model to incorporate concentrations in the effect-compartment (*Ce*) as the exposure term which drives the response (Eq. 6).

$$\frac{dCe}{dt}=\;ke0\text{*}Cp-ke0\text{*}Ce$$ (5)

$$E=E_{0}+\frac{E_{max}\text{*}Ce}{EC_{50}+Ce}$$ (6)

### *In Vivo* Exposure–Response Post Processing

To facilitate visualization of the exposure–response relationship across preclinical assays, the PD response in each assay was converted to percent maximum possible effect (%MPE) using Equation 7.

$$\text{\%}MPE=\frac{\left(max-baseline\right)-\left(max-observed\right)}{\left(max-baseline\right)}\text{*}100$$ (7)

To account for species differences in both plasma protein binding and intrinsic activity on the target, measured total plasma concentrations (*C*_*p,total*_) were multiplied by the measured species-specific fraction unbound in the plasma (*f*_*u,p*_) and then divided by the measured species-specific *in vitro* NaV1.7 *IC*_50_ value. Therefore, the exposure term *C* in Equations 3, 4 becomes a derived dimensionless scalar for unbound plasma concentration relative to *in vitro* potency as in Equation 8.

$$In\;Vivo/In\;Vitro\;Scalar=\;\frac{C_{p,total}\text{*}f_{u,p}}{in\;vitro\;IC_{50}}$$ (8)

Respectively, the ratio of unbound plasma concentration relative to *in vitro* potency required to achieve 50% effect in the preclinical *in vivo* assays can be derived by substituting *IC*_50_ or *EC*_50_ for *C*_*p,total*_ in Equation 8.

### Projection of Human Pharmacokinetics, Pharmacodynamics, and Efficacious Dose

The MK-2075 human PK parameters, namely, clearance and volume of distribution, were predicted from preclinical data using allometric scaling. The resulting PK parameters were then integrated with the preclinical PKPD model output to predict the dose required to achieve a target plasma concentration anticipated to be efficacious in the reduction of postoperative pain.

#### Pharmacokinetics

Human clearance and volume of distribution for MK-2075 were predicted using allometric scaling of two-compartment PK parameters obtained from rats, dogs, and monkeys, using an internal web-based application employing R script which enables incorporation and visualization of the impact of experimental uncertainty on the predicted dose and PK profile (Lindauer et al., 2014).

Elimination in preclinical species was balanced between metabolic and non–metabolic (renal or biliary excretion) pathways as determined from metabolite profiling of excreta in bile duct cannulated animals (unpublished data). Poor *in vitro* metabolic turnover and involvement of hepatic uptake and biliary efflux transporters in elimination resulted in an underestimation of *in vivo* metabolic clearance in preclinical species, precluding the use of *in vitro* biochemical data to predict human clearance (unpublished data). Therefore, allometric scaling was selected as the appropriate prediction method for human clearance. Unbound metabolic clearance was predicted by allometric scaling using Tang's coefficients (Tang et al., 2007). Non-metabolic unbound clearance (*CL*) and intercompartmental distribution CL (*CLD*) were scaled using fixed exponent allometry with the standard exponent of 0.75, and unbound body weight–normalized volumes (*V1* and *V2*) were scaled with the fixed exponent of 1.

#### Pharmacodynamics

The relationship between *in vitro* potency and *in vivo* potency determined from the mouse tail flick and rhesus thermode assays as described above was used to calculate a concentration target to achieve 50% effect in humans. The measured *in vitro* human NaV1.7 *IC*_50_ was multiplied by the derived *in vivo*/*in vitro* scalars and divided by the measured free fraction in the human plasma in order to translate to a total plasma concentration target for MK-2075 in humans. The biophase PKPD model used to characterize the exposure–response in the rhesus fMRI olfaction assay was subsequently used to predict the target modulation time course in humans at the anticipated clinical efficacious dose. Translation of this model to humans was accomplished by correcting for species differences in PK, *in vitro* potency, and plasma protein binding, while assuming that the kinetics of distribution to the target site and *in vitro* to *in vivo* translation of the NaV1.7 potency were the same between humans and rhesus monkeys. The mean and SE for individual parameter estimates, *ke0* and *EC*_50_, were used along with the correlation matrix to simulate uncertainty in the resulting PD profile within the human PK prediction application.

#### Efficacious Dose

The human PK prediction application generates a distribution of predicted human PK and PD parameters derived from the uncertainty in measured experimental data as described by Lindauer et al. (2014). Subsequently, 1,000 Monte-Carlo simulations were conducted at each dose level to generate the human concentration–time profiles while sampling from these parameter distributions, which results in a median-predicted profile with an associated confidence interval (CI) for each simulated dose level. Based on predicted human PK, preliminary simulations of various IV dose levels and infusion durations were explored to identify a dosing regimen which could achieve and sustain the PK target described above while also remaining below the exposure limits derived from preclinical toxicology studies. Ultimately, a dosing regimen consisting of a single IV infusion over a duration of 8 h was used for clinical dose and PD profile predictions.

## Results

### Benchmarking Preclinical Antinociceptive Response With Clinical SOC Analgesics

**FIGURE 2:**
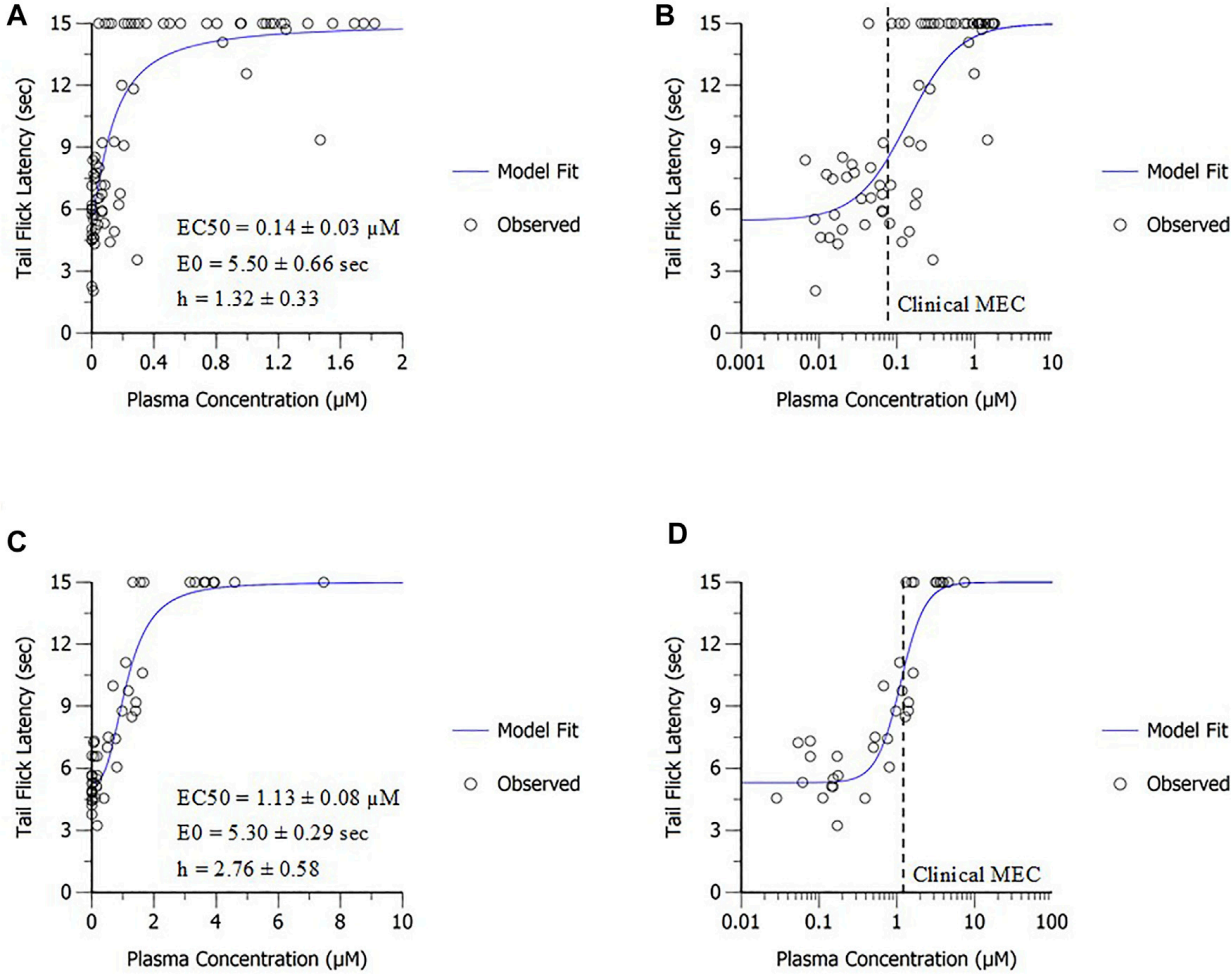
*In vivo* potency derived from the mouse tail flick latency assay for SOC postoperative pain therapeutics. Data are presented on **(A)** linear and **(B)** semi-log scale for morphine and **(C)** linear and **(D)** semi-log scale for tramadol.

Benchmarking antinociceptive response in preclinical assays for acute pain (mouse tail flick and rhesus thermode) with marketed SOC drugs was conducted to support the relevance of each assay for the target indication and provide a framework for PD target selection (Table 1). Total trough plasma concentrations following efficacious doses of the SOC analgesics used clinically to treat postoperative pain, namely, morphine, tramadol, and fentanyl, were obtained from the literature (Dahlstrom et al., 1982; Lehmann et al., 1990). The clinically efficacious concentrations were compared to the *in vivo* potency obtained from sigmoidal *E*_max_ modeling of the mouse tail flick latency data. Both SOC drugs, morphine and tramadol, achieved approximately 50% of the maximum possible effect in mouse tail flick latency at clinical efficacious concentrations (Figure 2), suggesting that the *EC*_50_ obtained in this assay for MK-2075 would be a reasonable exposure to the target in the clinic. Evaluation of morphine, tramadol, and fentanyl in the rhesus thermode heat withdrawal assay of nociception and correlation to clinical efficacious concentration has been described previously, and *IC*_50_ determined from this model aligned well with the clinical minimum efficacious concentration for postoperative pain (Vardigan et al., 2018). Therefore, concentrations of MK-2075 achieving a response of 50% inhibition in the rhesus thermode assay is expected to be an appropriate target for postoperative pain in humans.

**TABLE 1:** Comparison of preclinical potency and clinical minimum efficacious concentration (MEC) for SOC analgesics.

SOC analgesic	Clinical MEC^a^ (µM), mean (range)	Mouse tail flick *EC*_50_ (µM), mean ± SE
Morphine	0.074 (0.032–0.116)^b^	0.14 ± 0.03
Tramadol	1.092 (0.077–3.744)^c^	1.13 ± 0.08

a MEC is defined as the trough plasma concentration measured just prior to patient-controlled administration of a subsequent dose of analgesic.

b *Clinical Pharmacokinetics* 7: 266–279 (1982) (Dahlstrom, Tamsen, Paalzow, & Hartvig, 1982).

c *The Clinical Journal of Pain*, 6: 212–220 (1990) (Lehmann, Kratzenberg, Schroederbark, & Horrichshaermeyer, 1990).

### MK-2075 Preclinical PKPD and Translation to Clinical Exposure Targets

**FIGURE 3:**
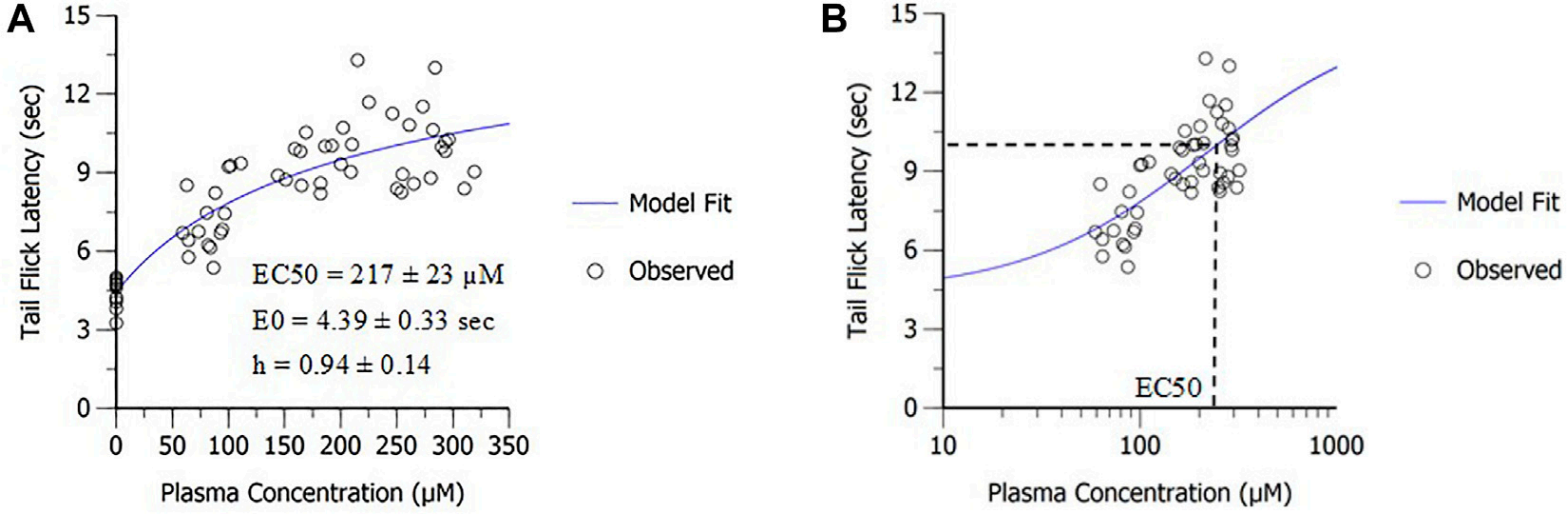
*In vivo* potency of MK-2075 was derived from the mouse tail flick latency assay as depicted in **(A)** linear scale and **(B)** semi-log scale.

**FIGURE 4:**
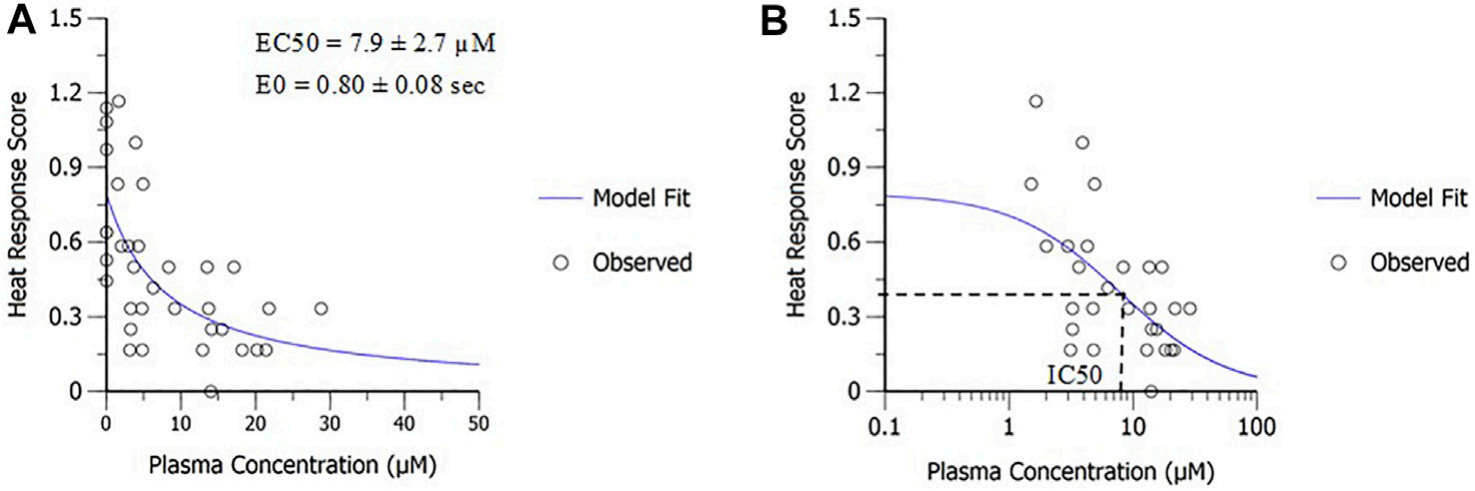
*In vivo* potency of MK-2075 derived from the rhesus thermode heat withdrawal assay at 46°C as depicted in **(A)** linear scale and **(B)** semi-log scale.

PD modeling was applied to determine *in vivo* potency of MK-2075 in preclinical nociception assays (mouse tail flick and rhesus thermode) and the NaV1.7 target modulation assay (fMRI olfaction). The sigmoidal *E*_max_ model fit of the MK-2075 exposure–response in mouse tail flick latency resulted in an *in vivo* potency (*EC*_50_) of 217 ± 23 µM (mean ± SE), baseline latency (*E*_0_) of 4.4 ± 0.3 s, and the Hill coefficient of 0.94 ± 0.14 (Figure 3). The inhibitory *E*_max_ model fit of the MK-2075 exposure–response in rhesus thermode heat withdrawal at a temperature of 46°C resulted in an *IC*_50_ of 7.9 ± 2.7 µM (mean ± SE) and a baseline response score (*E*_0_) of 0.80 ± 0.08 (Figure 4).

**FIGURE 5:**
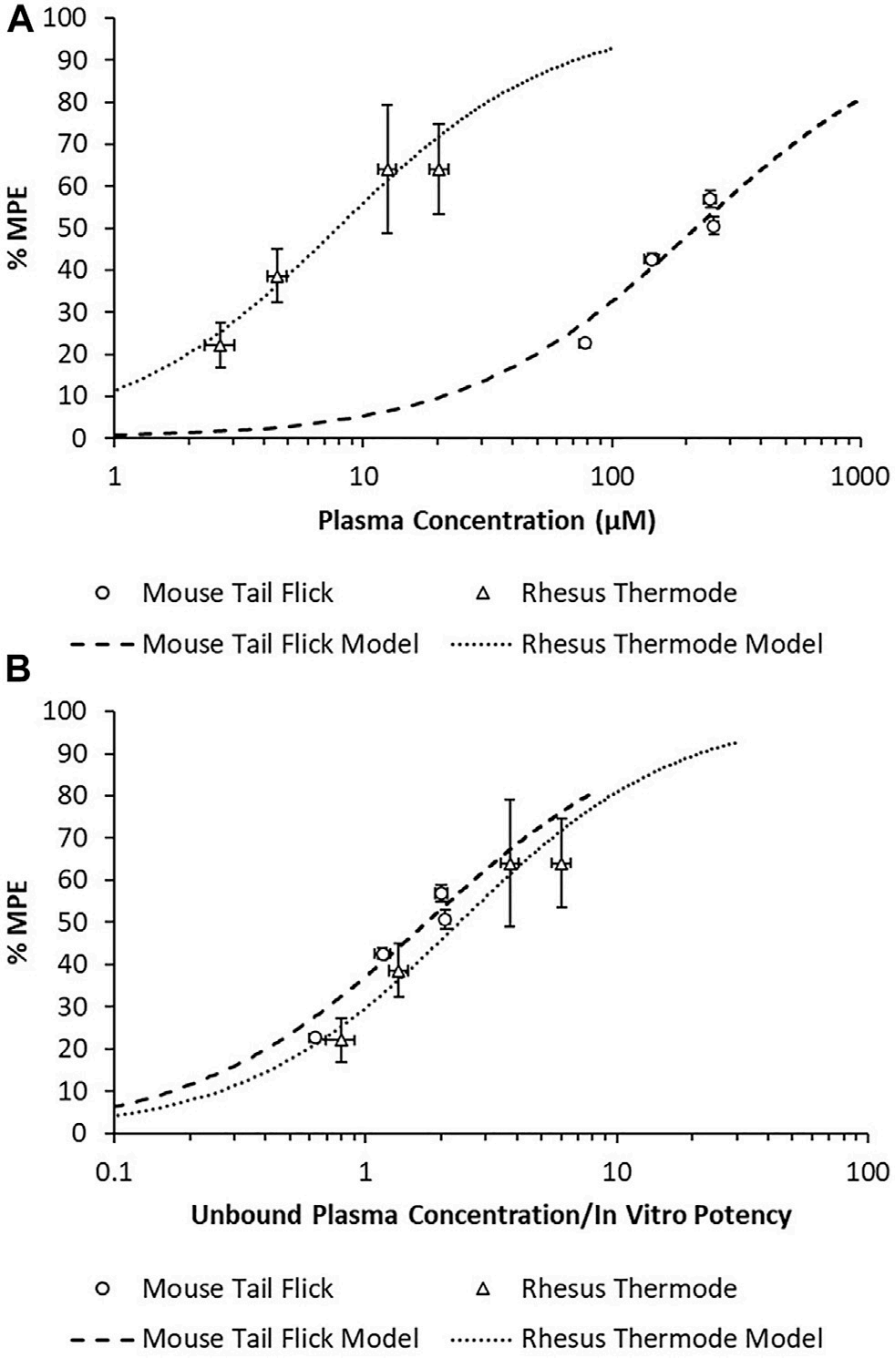
Overlay of MK-2075 exposure–response in the mouse tail flick latency and rhesus thermode heat withdrawal assays of nociception using **(A)** total plasma concentration and **(B)** unbound plasma concentration normalized by *in vitro* potency.

There was a substantial shift in the *in vivo* potency of MK-2075 obtained from the mouse tail flick assay relative to the rhesus thermode assay (Figure 5A). The expression of exposure–response in terms of the intrinsic potency–normalized unbound exposure results in improved alignment across species (Figure 5B) and a consistent ratio of unbound plasma concentrations (approximately twofold) over *in vitro* NaV1.7 *IC*_50_ to achieve 50% MPE in both the mouse and rhesus nociception assays (Table 2).

**TABLE 2:** Correction of MK-2075 *in vivo* potency for species-specific plasma protein binding and intrinsic NaV1.7 potency.

Species/assay	*In vivo EC*_50_ or *IC*_50_ (µM)	Plasma fraction unbound (*f*_*u,p*)_	*In vitro* NaV1.7 *IC*_50_ (µM)	$\frac{\text{In Vivo}}{\text{In Vitro}}\;$ Scalar
Mouse tail flick	217	0.026	3.228	1.75
Rhesus thermode	7.9	0.077	0.257	2.37

**FIGURE 6:**
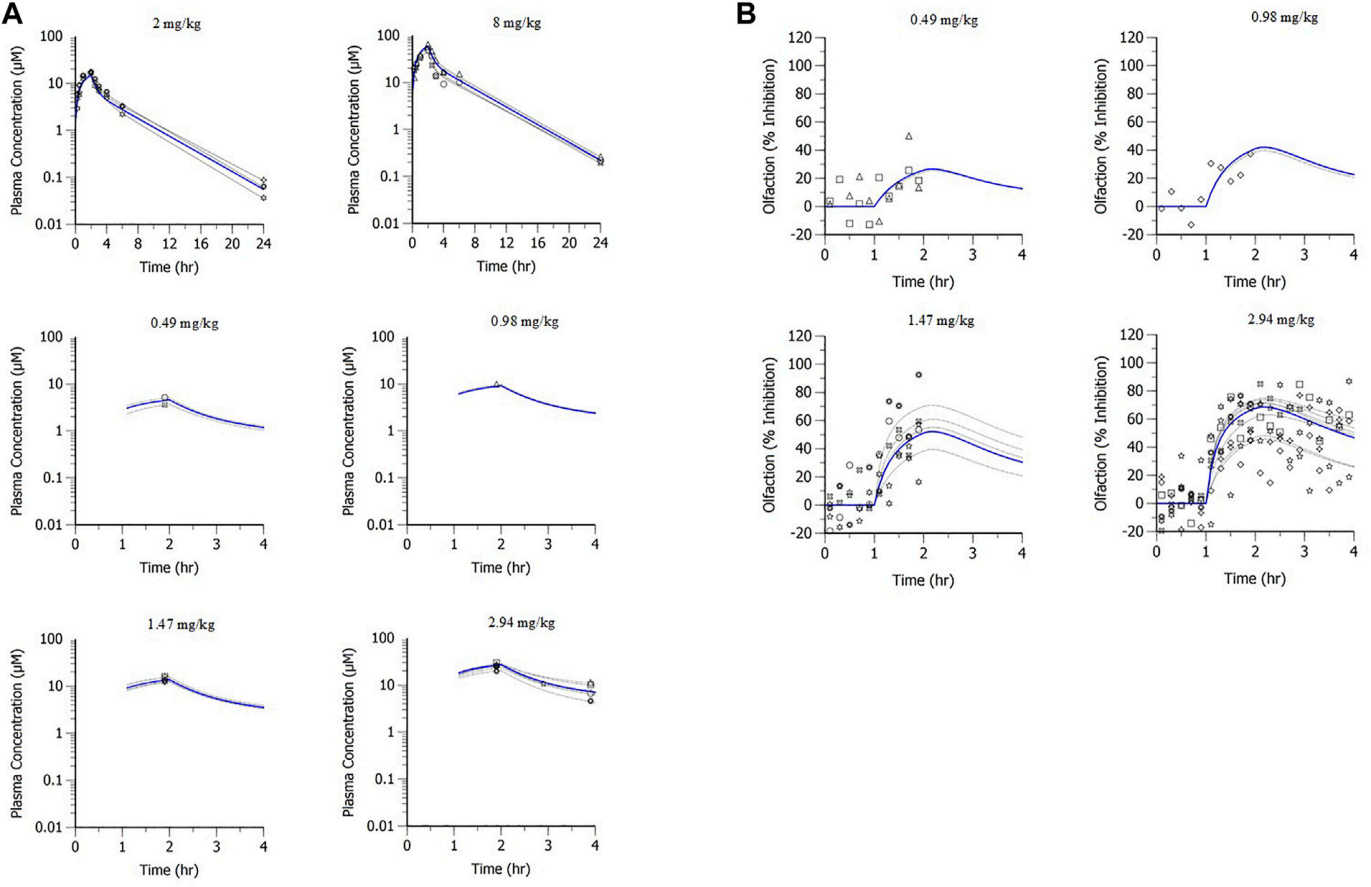
MK-2075 **(A)** PK and **(B)** PD of treatment-mediated inhibition of rhesus olfaction fMRI with two-compartment PK and biophase *E*_max_ PD models of fit.

**FIGURE 7:**
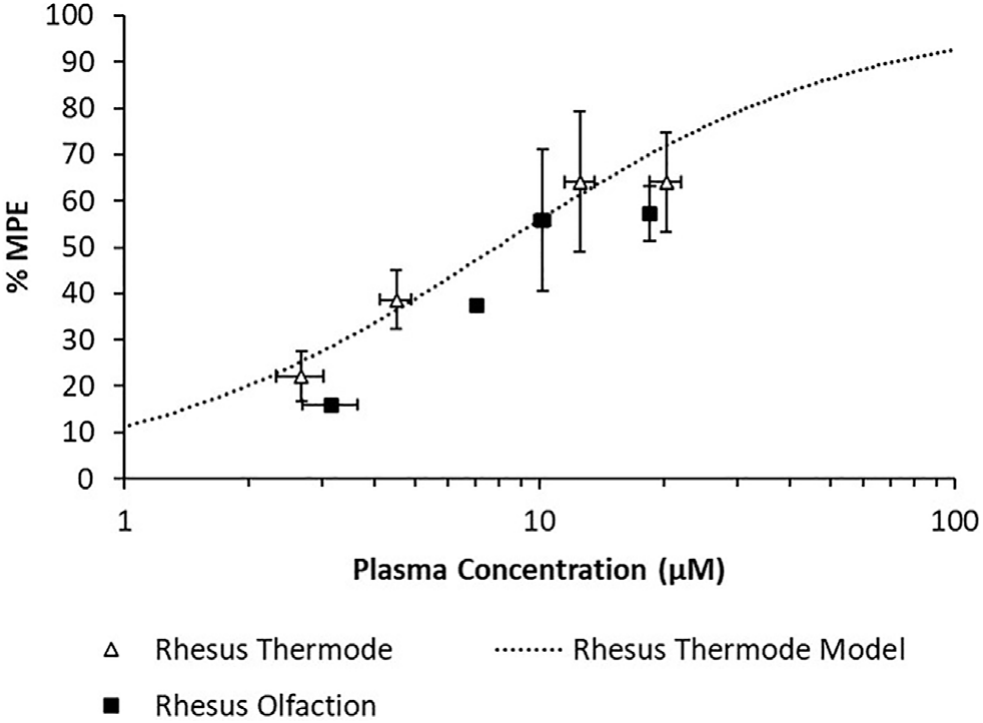
Overlay of observed rhesus olfaction exposure–response with model fit and observed rhesus thermode exposure–response.

A population PK model based on IV infusion in conscious rhesus monkeys adequately captured the MK-2075 concentrations observed in the anesthetized rhesus monkeys in fMRI olfaction studies (Figure 6A). Concentration–time predictions based on post-hoc PK analysis from anesthetized subjects enabled fitting of individual PD data. Incorporation of a biophase compartment to account for distributional delay corrected the hysteresis observed in the exposure–response data and adequately captured the time course of treatment-mediated inhibition of olfaction (Figure 6B). The resulting effect compartment *EC*_50_ was 10.3 ± 1.9 µM with a *ke0* of 2.1 ± 0.5 h^−1^. The measured exposure–response at the end of a 1-h infusion, when *Ce* and *Cp* were near equilibrium, aligns well with the results from the rhesus thermode assay (Figure 7).

The measured *in vitro* human NaV1.7 *IC*_50_ of MK-2075 was 0.149 µM, and a free fraction of 0.067 was measured in the human plasma. The application of the *in vivo*/*in vitro* scalars derived from the mouse tail flick and rhesus thermode assays to human *in vitro* NaV1.7 potency and correction for plasma protein binding results in a predicted clinical efficacious total plasma concentration of 3.9–5.3 µM (0.26–0.36 µM unbound, 1.75- to 2.37-fold over the *in vitro* NaV1.7 *IC*_50_) for MK-2075 in postoperative pain. The achievement of the upper bound of this predicted concentration range (5.3 µM) was selected as the clinical PK target and used to project efficacious human doses.

### Projection of MK-2075 Human Pharmacokinetics, Pharmacodynamics, and Efficacious Dose

**FIGURE 8:**
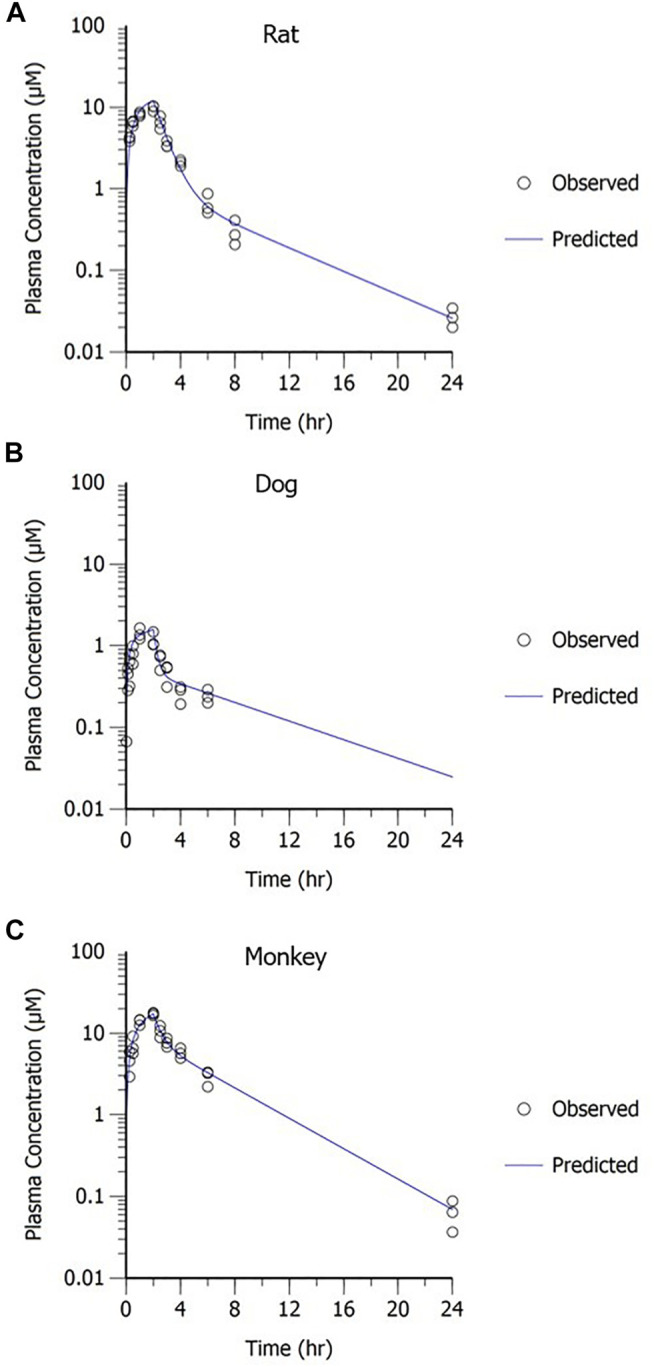
MK-2075 concentration–time profile in **(A)** rat, **(B)** dog, and **(C)** monkey with two-compartment PK model fit.

A two-compartment PK model with IV administration adequately captured the concentration–time profile observed in rats, dogs, and monkeys following a 2-h IV infusion (Figure 8). The resulting parameter estimates with SE and CV% are presented in Table 3. The consistent biphasic nature of the PK profile in preclinical species led us to assume that a two-compartment PK model structure would also be appropriate for humans.

**TABLE 3:** MK-2075 preclinical PK parameters from two-compartment model fit of concentration–time data in rat, dog, and monkey.

Species	Parameter	Estimate	SE	CV%
Wistar Han rat	V1 (L/kg)	0.072	0.006	8.3
	CL (L/hr/kg)	0.068	0.003	4.5
	V2 (L/kg)	0.066	0.008	12.3
	CLD (L/hr/kg)	0.013	0.002	16.8
Beagle dog	V1 (L/kg)	0.092	0.013	14.4
	CL (L/hr/kg)	0.149	0.009	5.8
	V2 (L/kg)	0.649	0.071	10.9
	CLD (L/hr/kg)	0.197	0.029	14.9
Rhesus monkey	V1 (L/kg)	0.096	0.010	11.0
	CL (L/hr/kg)	0.067	0.003	4.1
	V2 (L/kg)	0.133	0.015	11.6
	CLD (L/hr/kg)	0.073	0.020	26.8

The predicted human PK parameters from allometric scaling from rats, dogs, and monkeys, including uncertainty in the prediction due to variability in the measured input parameters, are presented in Table 4. The low clearance and long half-life contributed to a relatively long time to reach steady state with IV infusion administration. In early clinical studies, the concentration needs to be sustained above the PK target for a period of time sufficient to allow evaluation of the PD response. In order to achieve target concentrations for the desired duration (∼1 h) within the overall exposure and maximum concentration limits for safety, a long duration (8-h) IV infusion was required.

**TABLE 4:** Predicted human PK parameters of MK-2075 represented as median and 90% CI.

PK parameter	Median predicted value	90% confidence interval
CL (ml/min/kg)	0.402	0.368–0.440
V1 (L/kg)	0.063	0.055–0.071
V2 (L/kg)	0.130	0.114–0.148
CLD (ml/min/kg)	0.312	0.256–0.382
Terminal T1/2 (h)	9.5	8.9–10.1
Effective T1/2 (h)	5.5	5.2–6.0

**FIGURE 9:**
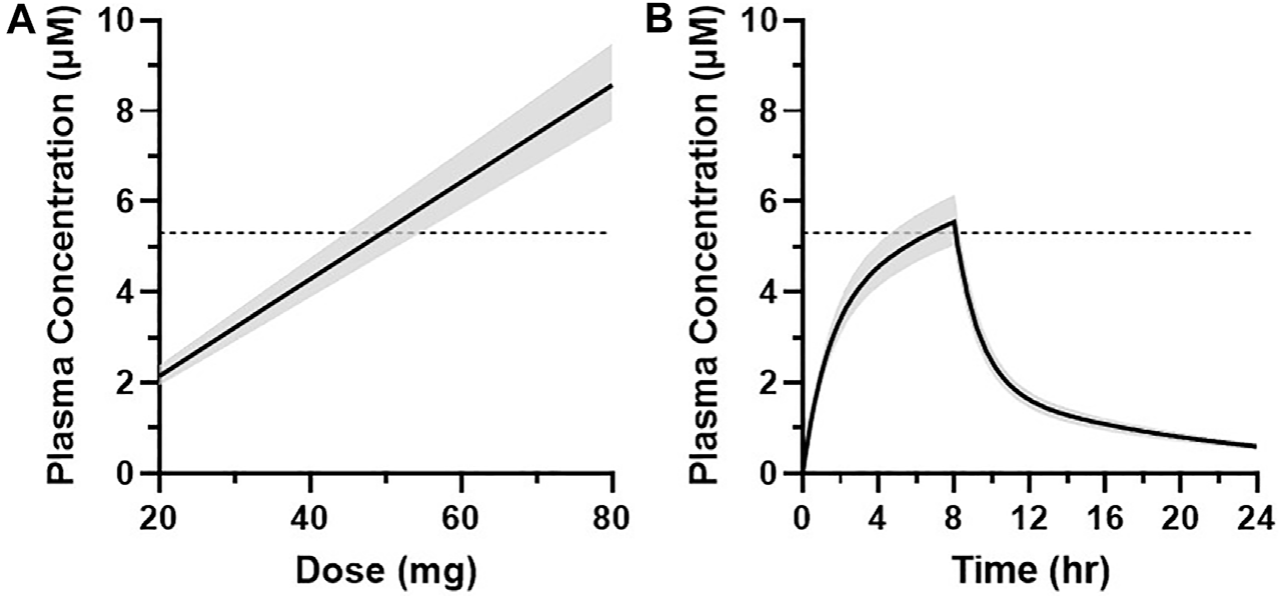
**(A)** Graphical representation of the simulated plasma concentration (median ± 90% CI) at 7 h after the start of infusion at multiple dose levels in humans. **(B)** Simulated MK-2075 plasma concentration over time (median ± 90% CI) at a predicted dose of 50 mg infused intravenously over 8 h. Dashed horizontal line represents the target plasma concentration of 5.3 µM.

**FIGURE 10:**
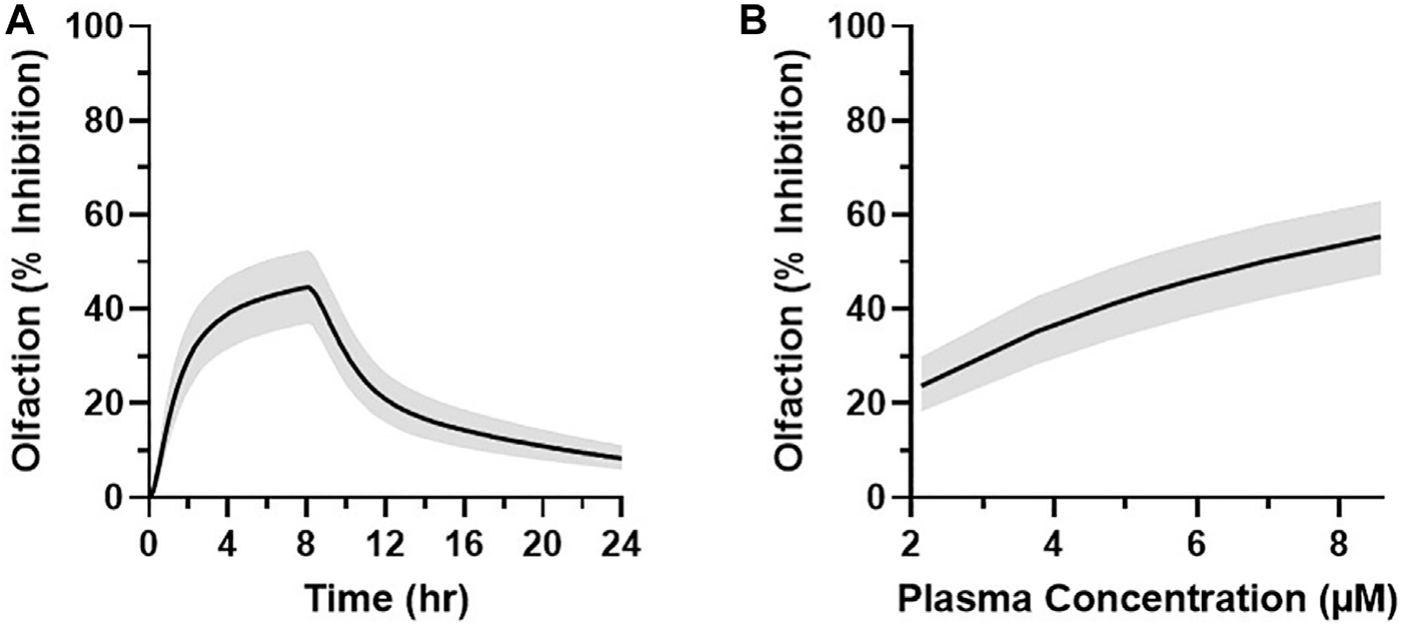
**(A)** Simulated MK-2075 percent olfactory response inhibition over time (median ± 90% CI) at a predicted dose of 50 mg infused intravenously over 8 h in humans. **(B)** Graphical representation of the simulated olfaction inhibition response (median ± 90% CI) at 7 h after the start of infusion as a function of the simulated plasma concentration of MK-2075 at 7 h after doses of 20, 35, 45, 50, 55, 65, and 80 mg.

The projected dose to achieve the target median plasma concentration of 5.3 µM for at least 1 h was 50 mg over an 8-h infusion with a 90% CI of 45–55 mg (Figure 9A). This dose level corresponds to the median total plasma concentrations of 5.3–5.5 µM between 7 and 8 h and an AUC_0–24h_ of 56 μM·h (90% CI of 51–61 μM·h) (Figure 9B). Translation of the PKPD modeling results from rhesus olfaction indicates that the anticipated median target modulation achievable within the last hour of an 8-h infusion at 6.25 mg/h (50 mg total dose) in humans would be 43% inhibition (90% CI of 37–53%) (Figure 10A). Doses ranging from 20 to 80 mg infused over 8 h are projected to achieve average total plasma concentrations of 2.1–8.6 µM in the last hour of infusion and anticipated to produce 24–55% average inhibition of olfaction (Figure 10B).

## Discussion

Based on clinical benchmarking with marketed SOC analgesics, concentrations eliciting 50% inhibition in the rhesus thermode heat withdrawal assay and the mouse tail flick are associated with postoperative pain mitigation in the clinic, and 50% inhibition is therefore a reasonable PD target to test the effectiveness of inhibition of NaV1.7 in acute pain. The quantitative models of MK-2075 exposure–response with normalization for species differences in plasma protein binding and *in vitro* potency established an understanding of the *in vitro* to *in vivo* translation of target potencies. This enables translation of PD response in preclinical species to humans when species differences exist in target potency, plasma fraction unbound, and PK. Integration of *in vitro* human NaV1.7 potency and plasma protein binding into these models provided a prediction of the total plasma concentration PK target of 5.3 µM (0.36 µM unbound, 2.4-fold over the *in vitro* NaV1.7 *IC*_50_) for MK-2075 in postoperative pain. Allometric scaling of preclinical PK parameters and translation of a biophase PKPD model based on rhesus olfaction with correction for species differences in potency and plasma protein binding was used to predict human PK, PD, and dose regimen. The anticipated target modulation achieved by 8 h following an infusion of 50 mg total dose to humans was projected to be ∼40–50% inhibition. The results of this model-based analysis of preclinical PKPD and translation to human PKPD and dose projections described herein were used to identify a compound with sufficient PK and safety profile to test the NaV1.7 inhibition mechanism in humans and aid the clinical study design and inform decision criteria in the early clinical development plan.

The quantitative translation of preclinical PKPD and antinociceptive activity to anticipated human PKPD and efficacious dose requires some assumptions and therefore will have some inherent limitations. A critical assumption in the benchmarking analysis is that there are no meaningful species difference in intrinsic potency for each of these SOC compounds against their biological target. Since the benchmarking analysis was conducted with drugs acting on a different biological target than MK-2075, an assumption was required that the observed translation of response in the preclinical nociception assay to clinical efficacy will be independent of the mechanism and is therefore applicable to the inhibition of NaV1.7. The potential for potency differences depending on the type of pain stimulus must also be considered. Bankar et al. (2018) demonstrated a shift in potency between a thermal stimulus assay using a hot plate and the traditional assays for inflammatory or neuropathic pain for NaV1.7 antagonists. Multiple structural analogs of MK-2075 have been evaluated in a mouse formalin paw assay demonstrating 90% inhibition of behavioral response to formalin stimulus at unbound concentrations approximately equal to the *in vitro* potency (Roecker et al., 2021). This represents a substantial leftward shift from a value of unbound *EC*_50_ approximately twofold over *in vitro* potency for thermal stimulus in the mouse tail flick assay, and it is consistent with the results obtained by Bankar et al., suggesting that NaV1.7 inhibitors may require greater target engagement to elicit antinociceptive effects in assays of acute nociception compared to assays of inflammatory/persistent nociception.

Another important consideration in the interpretation of the mouse tail flick latency results is a potential for species-specific selectivity profiles. While MK-2075 has robust selectivity for human NaV1.7 over the other isoforms such as NaV1.6 and NaV1.5 (>500-fold, manuscript in preparation), the selectivity profile for the mouse isoforms of the sodium ion channel has not been evaluated. Given the substantial shift in NaV1.7 potency between humans and mice, it is possible that selectivity for the NaV1.7 isoform is reduced in rodents. Therefore, at the high unbound concentrations of MK-2075 required for inhibition in the mouse tail flick assay, pharmacological activity at other NaV isoforms cannot be ruled out.

The direct-effect PD models selected for fitting of mouse tail flick latency and rhesus thermode heat withdrawal data assume that there is no hysteresis in the PKPD relationship. While graphical analysis of the mouse tail flick latency measured at different time points with structural analogs supports this assumption, the exposure–response data in rhesus thermode studies consisted of only a single time point concentration measurement at study termination, prohibiting the evaluation of potential for hysteresis. A key assumption in the rhesus olfaction PKPD model is that hysteresis is presumed to be a result of slightly delayed distribution to the target site due to the poor permeability of MK-2075. This assumption was informed by PKPD analysis with other NaV1.7 inhibitors, indicating that the extent of hysteresis is compound dependent and can likely be attributed to poor permeability causing slow distribution into the nerves (Ballard et al., 2020). The distribution rate constant (*ke0*) for the site of action in humans is assumed to be equivalent to that in rhesus monkeys.

Lastly, inhibition of olfaction as measured by fMRI is anticipated to be a relevant marker of target modulation based on the genetic evidence of both anosmia and loss of pain sensation in individuals with the NaV1.7 LOF mutation. While concordance was observed between inhibition of olfaction and heat withdrawal score in the rhesus assays with MK-2075, it is reasonable to consider that the magnitude of restriction at the blood–nerve barrier could be different at the peripheral nerves in the forearm than at the olfactory epithelium. There are structural differences identified in the blood–nerve barrier along different regions of the olfactory receptor neurons as well as differences in the tight junction protein occludin relative to the blood–nerve barrier in typical peripheral nerves such as the sciatic nerve (Hussar et al., 2002) (Tserentsoodol et al., 1999). A quantitative relationship between treatment-mediated inhibition of olfaction and reduction in pain sensation has not yet been established in the clinic. A recent clinical study with a NaV1.7 inhibitor, GDC-0276, monitored reduced sense of smell (hyposmia) as a potential biomarker of on-target pharmacology (Rothenberg et al., 2019). While a couple of incidents of hyposmia were reported, the authors conclude that due to a lack of an exposure-related pattern, the findings do not support impaired sense of smell as a biomarker. However, it is unclear if the relatively low unbound plasma concentrations obtained in this study achieved sufficient target engagement at the site of action to inhibit olfaction. The authors themselves acknowledge that further study will be needed to define the exposures required to achieve on-target PD effects.

In summary, MK-2075 administered as a continuous IV infusion of 50 mg over an 8-h duration is projected to achieve sufficient NaV1.7 target engagement to further evaluate the potential of this target for treatment of pain indications. Benchmarking the desired preclinical antinociceptive response against clinical SOC for acute postoperative pain and establishing the *in vitro* to *in vivo* potency relationship for MK-2075 in both the mouse and rhesus assays afforded a data-driven PKPD target for the clinic. The quantitative integration of intrinsic potency against the human NaV1.7 target, predicted unbound exposure in the plasma, and distribution to the site of action using mathematical PKPD modeling enabled projection of clinically efficacious dose and simulation of anticipated PD profile of olfaction as a potential target modulation biomarker.

## Data Availability

The raw data supporting the conclusions of this article will be made available by the authors, without undue reservation.

## References

[B1] Ballard J. E. Pall P. Vardigan J. Zhao F. Holahan M. A. Kraus R. (2020). Application of Pharmacokinetic-Pharmacodynamic Modeling to Inform Translation of *In Vitro* NaV1.7 Inhibition to *In Vivo* Pharmacological Response in Non-human Primate. Pharm. Res. 37, 181. 10.1007/s11095-020-02914-9 32888082 PMC7473964

[B2] Bankar G. Goodchild S. Howard S. Nelkenbrecher K. Waldbrook M. Dourado M. (2018). Selective NaV1.7 Antagonists With Long Residence Time Show Improved Efficacy Against Inflammatory and Neuropathic Pain. Cel Rep. 24, 3133–3145. 10.1016/j.celrep.2018.08.063 30231997

[B3] Bueters T. Gibson C. Visser S. (2015). Optimization of Human Dose Prediction by Using Quantitative and Translational Pharmacology in Drug Discovery. Future Med. Chem. 7 (17), 2351–2369. 10.4155/fmc.15.143 26599348

[B4] Dahlstrom B. Tamsen A. Paalzow L. Hartvig P. (1982). Patient-Controlled Analgesic Therapy .4. Pharmacokinetics and Analgesic Plasma-Concentrations of Morphine. Clin. Pharmacokinet. 7 (3), 266–279. 10.2165/00003088-198207030-00006 7094501

[B5] Dib-Hajj S. Cummins T. Black J. Waxman S. (2007). From Genes to Pain: Na(v)1.7 and Human Pain Disorders. Trends Neurosciences. 30 (11), 555–563. 10.1016/j.tins.2007.08.004 17950472

[B6] Dick I. Brochu R. Purohit Y. Kaczorowski G. Martin W. Priest B. (2007). Sodium Channel Blockade May Contribute to the Analgesic Efficacy of Antidepressants. J. Pain. 8 (4), 315–324. 10.1016/j.jpain.2006.10.001 17175203

[B7] Hussar P. Tserentsoodol N. Koyama H. Yokoo-Sugawara M. Matsuzaki T. Takami S. (2002). The Glucose Transporter GLUT1 and the Tight Junction Protein Occludin in Nasal Olfactory Mucosa. Chem. Senses. 27 (1), 7–11. 10.1093/chemse/27.1.7 11751462

[B8] Jansson-Löfmark R. Hjorth S. Gabrielsson J. (2020). Does *In Vitro* Potency Predict Clinically Efficacious Concentrations? Clin. Pharmacol. Ther. 108 (2), 298–305. 10.1002/cpt.1846 32275768 PMC7484912

[B9] Kraus R. Zhao F. Pall P. Zhou D. Vardigan J. Danziger A. (2021). Na(v)1.7 Target Modulation and Efficacy Can Be Measured in Nonhuman Primate Assays. Sci. Translational Med. 13. 10.1126/scitranslmed.aay1050 34011626

[B10] Lehmann K. Kratzenberg U. Schroederbark B. Horrichshaermeyer G. (1990). Postoperative Patient-Controlled Analgesia With Tramadol - Analgesic Efficacy and Minimum Effective Concentrations. Clin. J. Pain. 6 (3), 212–220. 10.1097/00002508-199009000-00008 2135015

[B11] Lindauer A. Y. Guo K. Lee B. Ivashina J. Deshmukh S. (2014). A Tool for First-In-Human PK Prediction Incorporating Experimental Uncertainty. PAGE. 23. Available at: Abstr 3171. www.page-meeting.org/?abstract=3171

[B12] Morgan P. Van Der Graaf P. Arrowsmith J. Feltner D. Drummond K. Wegner C. (2012). Can the Flow of Medicines Be Improved? Fundamental Pharmacokinetic and Pharmacological Principles Toward Improving Phase II Survival. Drug Discov. Today. 17 (9-10), 419–424. 10.1016/j.drudis.2011.12.020 22227532

[B13] Moulin D. Boulanger A. Clark A. Clarke H. Dao T. Finley G. (2014). Pharmacological Management of Chronic Neuropathic Pain: Revised Consensus Statement From the Canadian Pain Society. Pain Res. Management. 19 (6), 328–335. 10.1155/2014/754693 PMC4273712 25479151

[B14] Roecker A. J. Layton M. E. Pero J. E. Kelly M. J. Greshock T. J. Kraus R. L. (2021). Discovery of Arylsulfonamide Nav1.7 Inhibitors: IVIVC, MPO Methods, and Optimization of Selectivity Profile. ACS Med. Chem. Lett. 12, 1038–1049. 10.1021/acsmedchemlett.1c00218 34141090 PMC8201757

[B15] Rothenberg M. Tagen M. Chang J. Boyce-Rustay J. Friesenhahn M. Hackos D. (2019). Safety, Tolerability, and Pharmacokinetics of GDC-0276, a Novel Na(V)1.7 Inhibitor, in a First-In-Human, Single- and Multiple-Dose Study in Healthy Volunteers. Clin. Drug Invest. 39 (9), 873–887. 10.1007/s40261-019-00807-3 31172446

[B16] Tanelian D. L. Victory R. A. (1995). Sodium Channel-Blocking Agents: Their Use in Neuropathic Pain Conditions. Pain Forum. 4 (2), 75–80. 10.1016/s1082-3174(11)80001-2

[B17] Tang H. Hussain A. Leal M. Mayersohn M. Fluhler E. (2007). Interspecies Prediction of Human Drug Clearance Based on Scaling Data From One or Two Animal Species. Drug Metab. Disposition. 35 (10), 1886–1893. 10.1124/dmd.107.016188 17646280

[B18] Tserentsoodol N. Shin B.-C. Koyama H. Suzuki T. Takata K. (1999). Immunolocalization of Tight Junction Proteins, Occludin and ZO-1, and Glucose Transporter GLUT1 in the Cells of the Blood-Nerve Barrier. Arch. Histology Cytol. 62 (5), 459–469. 10.1679/aohc.62.459 10678575

[B19] Vardigan J. Houghton A. Lange H. Adarayan E. Pall P. Ballard J. (2018). Pharmacological Validation of a Novel Nonhuman Primate Measure of thermal Responsivity With Utility for Predicting Analgesic Effects. J. Pain Res. 11, 735–741. 10.2147/JPR.S152879 29692626 PMC5903490

[B20] Weiss J. Pyrski M. Jacobi E. Bufe B. Willnecker V. Schick B. (2011). Loss-of-Function Mutations in Sodium Channel Na(v)1.7 Cause Anosmia. Nature. 472, 186–190. 10.1038/nature09975 21441906 PMC3674497

[B21] Wong H. Bohnert T. Damian-Lordache V. Gibson C. Hsu C.-P. Krishnatry A. S. (2017). Translational Pharmacokinetic-Pharmacodynamic Analysis in the Pharmaceutical Industry: an IQ Consortium PK-PD Discussion Group Perspective. Drug Discov. Today. 22, 1447–1459. 10.1016/j.drudis.2017.04.015 28476536

[B22] Zhao F. Holahan M. A. Houghton A. K. Hargreaves R. Evelhoch J. L. Winkelmann C. T. (2015). Functional Imaging of Olfaction by CBV fMRI Inmonkeys: Insight into the Role of Olfactory Bulb in Habituation. NeuroImage. 106, 364–372. 10.1016/j.neuroimage.2014.12.001 25498426

